# ACVIM‐Endorsed Statements: Consensus statements, evidence‐based practice guidelines and systematic reviews

**DOI:** 10.1111/jvim.16869

**Published:** 2023-10-03

**Authors:** ACVIM Consensus Statement Taskforce, Kenneth W. Hinchcliff, Paul S. Morley, Stephen P. DiBartola, Sandra D. Taylor, Karyn A. Harrell

**Affiliations:** ^1^ Melbourne Veterinary School University of Melbourne, and Trinity College Parkville Victoria Australia; ^2^ Texas A&M University VERO Program, Department of Clinical Sciences College Station Texas USA; ^3^ College of Veterinary Medicine The Ohio State University Columbus Ohio USA; ^4^ College of Veterinary Medicine, Department of Veterinary Clinical Sciences, West Lafayette Purdue University Indiana USA; ^5^ College of Veterinary Medicine North Carolina State University Raleigh North Carolina USA

**Abbreviations**
ACVIM: American College of Veterinary Internal Medicine
AES: ACVIM‐Endorsed Statement
BoR: Board of Regents
COI: conflict of interest
ERC: Education and Research Committee
GRADE: Grading of Recommendations, Assessment, Development, and Evaluation
PRISMA: Preferred Reporting Items for Systematic Reviews and Meta‐Analyses

The American College of Veterinary Internal Medicine (ACVIM) has commissioned groups of experts to produce “Consensus Statements” since 2000 to provide veterinarians with information about topics important to ACVIM Diplomates and the veterinary profession. The ACVIM Consensus Statements have been relatively succinct statements intended to guide decision‐making about diagnosis, management or treatment of diseases of animals. During the past 20 years, Consensus Statements have evolved and several versions of guidelines have been developed by the ACVIM Board of Regents (BoR) and staff to facilitate selection of the topics, identify panel members, and determine logistics related to format and dissemination of the statements. The general purpose of these guidelines has been to clarify the responsibilities of the Chair and members of the panel, define the length of the statement, set timelines, and establish a financial agreement with panelists. Little attempt has been made to define how ACVIM Consensus Statements are prepared and the manner in which they consider the relevant literature and evaluate and report on evidence.

The medical and veterinary communities increasingly rely on evidence‐based statements on a particular topic for guidance and counsel on decisions relating to management of diseases.^1^, ^2^ There are several conventional formats for these statements, including consensus statements, practice guidelines, systematic reviews and position statements.^3^, ^4^, ^5^, ^6^, ^7^, ^8^ Recently, scoping reviews have become more common and are gaining some traction in the veterinary literature.^9^ The methodology underlying each of these types of statements differs, but what all have in common is a well‐described and accepted methodology for  objectively considering and reporting the amount and quality of evidence. For example, the Grading of Recommendations, Assessment, Development and Evaluation (GRADE) approach is now widely used for developing practice (and other) guidelines, Cochrane prescribes a particular methodology for undertaking evidence‐based reviews, Preferred Reporting Items for Systematic Reviews and Meta‐Analyses (PRISMA) defines how systematic reviews should be carried out, and the Delphi method is widely accepted for development of consensus statements.^4^, ^5^, ^7^, ^8^, ^10^, ^11^, ^12^, ^13^, ^14^, ^15^ Scoping reviews are not designed or intended to produce a critically appraised and synthesized result/answer to a particular question and are therefore not included as an ACVIM‐Endorsed Statement (AES).^9^ Narrative reviews are common in both the human and veterinary medical literature and there is a proposed methodology for evaluating their quality,^16^ but they are not considered sufficient to provide a rigorous, evidence‐based assessment of a topic.^17^ Narrative reviews are not listed in the Oxford Centre for Evidence‐Based Medicine hierarchy of evidence.^17^ There is increasing recognition of the need for clear guidelines for the development of expert statements.^1^

The variety of formats for these evidence‐based statements reflects that the underlying reason for commissioning a statement dictates its format. One size does not fit all, and such is the case with ACVIM Consensus Statements as reflected by the variety of formats found in the 45 Consensus Statements published through the end of 2020. Most (n = 34, 76%) of these Consensus Statements are narrative reviews, although recently some statements have followed GRADE, Delphi or similar methodologies, likely reflecting increased recognition that ACVIM Consensus Statements should be developed using explicit and transparent criteria and methodologies.

Of the 45 ACVIM Consensus Statements published between 2000 and 2020, only 13/45 (29%) describe the methodology used to develop the statement, 11 (24%) define particular questions to be addressed in the statement, 9 (20%) utilize descriptions of levels of evidence, and 4 (9%) address a limited number of questions (ie, narrowly define the topic) yet 20 (44%) provide specific recommendations. Clearly, guidelines in effect to date have not resulted in ACVIM Consensus Statements consistently conforming to contemporary standards for such expert statements.

The ACVIM Consensus Statements make valuable contributions to the veterinary literature, providing practicing specialists and nonspecialists with recommendations developed by experts in the field and are consistently the most widely read of all articles published in the Journal of Veterinary Internal Medicine (based on downloads and citations). The ACVIM Consensus Statements are increasingly recognized as being an important source of expert advice and, as such, reflect well on the College. However, quality standards for such statements are increasing, and we believe that it is time that the College consider developing more uniform and rigorous practices and policies for creation of these statements. Doing so will ensure that readers of such expert statements are confident of the quality of the statement and that the statement reflects well on the ACVIM and the authors.

## 1 ROLE OF THE ACVIM

The ACVIM supports the production of materials intended to assist clinicians and others in assessing the best available or optimal approach to providing veterinary care of animals or in understanding aspects of animal disease. These have been referred to as ACVIM Consensus Statements.

One of the roles of the ACVIM is to identify appropriate topics and to ensure that these Consensus Statements are uniformly reliable and of high quality. To achieve this purpose, the ACVIM must ensure that topics are timely and relevant, panels are appropriately comprised of experts who are free of conflicts of interest, methodology is rigorous and transparent, and that there is a process for review of the draft statement.

## 2 ACVIM‐ENDORSED STATEMENTS

We proposed, and the Board of Regents accepted, that subsequent expert statements endorsed by the ACVIM be collectively referred to as AESs and that they be developed using specific methodologies. The roles of the Board of Regents and the Education and Research Committee may be modified from that proposed below to align with changes in the functions of the Board and Board officers.

The term “Consensus Statement” previously covered all such expert statements, but in the current terminology “consensus statement” has a much narrower definition. We propose that AES follow one of three formats:

1. Consensus statements (as more narrowly defined below).
2. Evidence‐based practice guidelines.
3. Systematic reviews.

Narrative reviews, scoping reviews, and position papers are not considered suitable forms of AES. A narrative review provides a summary of current and relevant literature, but with few or limited comments on the quality of evidence and no formal recommendations for action. Importantly, they do not use PRISMA methodology.^2^, ^15^, ^16^ Scoping reviews do not seek to answer a particular question or questions, but are intended to identify certain characteristics or concepts in papers or studies, and examine the mapping, reporting or discussion of these characteristics or concepts.^9^ A position paper provides the view of an individual or group on a particular issue, is not developed with any defined methodology, and is in many cases an opinion piece.

The appropriate format for an AES will vary depending on the topic, the available published evidence, and the intent of the statement. The AES can be developed using one of three approved methodologies. The methodology chosen for a particular AES will be decided as part of the process for selecting a topic, convening a panel, and developing specific questions.

Regardless of the format, the AES should:

1. Have a defined, focused scope and address a topic by considering a series of unambiguous, succinct, and precise questions.
2. Use methodology appropriate for the topic and tailored to meet the requirements of the particular topic and questions.
3. Clearly describe the methodology, which will be different for each of the three types of statements.
4. Be well‐written, concise, precise reports that conform to the Journal of Veterinary Internal Medicine format and editorial guidelines.

## 3 TYPES OF ACVIM‐ENDORSED STATEMENTS

### 3.1 Consensus statement

Consensus statements are always developed using the Delphi method and reflect the collective opinions of a group of content experts. The opinions of the experts will be based on their individual expertise, experience, and knowledge of the literature. Consensus is tested and demonstrated using prescribed methodology to identify areas of agreement and disagreement within the group of experts.^14^, ^18^

Consensus statements are applicable to situations where evidence is limited or lacking, but where there is the opportunity to reduce uncertainty and improve quality of care.^6^, ^14^ Consensus statements take advantage of the collective wisdom of a group of experienced clinicians and researchers, substituting expert opinion for objective fact. The opinion of the experts is based on their individual experience and knowledge of the relevant scientific literature.

A consensus statement is applicable to situations where the evidence base is of insufficient quantity, quality, or is too varied for development of practice guidelines.

Consensus statements are characterized by:

1. A topic that is not amenable to consideration using an evidence‐based practice guideline methodology or systematic review.
2. Development using the Delphi methodology of iterative, systematic consideration and voting, not necessarily involving face‐to‐face meetings.
3. A core process that considers a series of statements, established a priori, upon which consensus is or is not achieved, as defined by previously agreed criteria for consensus.

An example of development of clinical consensus statements is the process used by otolaryngologists in human medicine.^14^

Consensus statements should not be based on a general statement of a topic (eg, “Asthma in cats”) but should be more narrowly focused. In this example, a more narrowly defined topic would be “Treatment of asthma in cats,” which addresses the specific statements: (1) inhaled corticosteroids are indicated; (2) betamethasone is the optimal inhaled corticosteroid; (3) oral corticosteroids are less effective than inhaled corticosteroids; and (4) beta‐adrenergic bronchodilators are indicated. When considering the topic and its breadth, the available length of the final document should be considered. Statements should be concise and precise. Topics should be defined narrowly enough so as to be addressed within the limitations on length of the statement.

### 3.2 Evidence‐based practice guidelines

Evidence‐based practice guidelines (“guidelines”) guide provision of current best practice care to animals and are ideally based on a rigorous, methodical, evidenced‐based review of the relevant literature. Guidelines have become increasingly important and useful to practicing clinicians given the increasing availability of novel or innovative treatments, interventions, or diagnostic methodologies and large quantity of scientific literature.

Guidelines should have a consistent and transparent method of development, be of uniformly high quality, and be free of bias or conflict of interest by the developers of the guidelines. High quality guidelines recommend a course of action based on a comprehensive and systematic review, grading of the evidence, and explicit comparison of the benefits and harms of a given test or treatment, such as achieved by the GRADE (Grading of Recommendations, Assessment, Development, and Evaluation) process.^4^, ^5^, ^7^, ^8^, ^14^ The Institute of Medicine provides a detailed user guide for preparation of guidelines as does the Cochrane website and Cochrane handbook on preparation of systematic reviews and guidelines, which also includes access to software for preparing guidelines compliant with GRADE (https://gradepro.org/product/#about; Table 1).^7^, ^10^, ^11^

**TABLE 1:** Institute of Medicine standards for trustworthy guidelines.

Domain‐specific standards	
Transparency	Funding and development process should be described and made public
Conflicts of interest (COI)	All COIs (including potential or perceived conflicts) should be disclosed
	Chair/Co‐Chair should not have COI
	Majority of panel should not have COI
	Members with financial conflicts should divest
	Funders, broadly defined, should not play a role in development of the guidelines
	Management of COI should be transparent and explicit; simple declaration is insufficient
Panel composition	Panel should be multidisciplinary
Literature review	Evidence synthesis should adhere to accepted standards for trustworthy reviews and development of guidelines
Grading of recommendations	Systematic approach should be used to summarize benefits and harms, rate the quality of the evidence, grade the strength of recommendations, incorporate values and preferences, and acknowledge differences in opinion (eg, GRADE), or to develop consensus
Articulation of recommendations	Use standardized format, such as “population, intervention, comparator, outcomes (ie, PICO)”
Review process	Review of draft document should be confidential (anonymous)—if peer reviewed
	Review should be performed by diverse stakeholders
	Draft should be available for public comment
Assessment of currency and updating	Dates of time line for preparation of the AES and publication should be stated

An important component of a Guideline is the Summary of Findings table. A Summary of Findings table presents the main findings of a review in a transparent and simple tabular format. In particular, the table provides key information concerning the quality of evidence, the magnitude of effect of the interventions examined, and the sum of available data on the main outcomes.^7^

### 3.3 Systematic reviews

Systematic reviews are important to veterinarians because they provide an objective assessment of existing data to assist with decisions about clinical practice, can provide rationale and justification for research, and are used as the basis for evidence‐based practice guidelines. The usefulness of a systematic review depends on the methodology and the clarity of reporting.

A systematic review is a review of a clearly formulated question that uses systematic and explicit methods to identify, select, and critically appraise relevant research, and to collect and analyze data from the studies that are included in the review. Statistical methods (meta‐analysis) may or may not be used to analyze and summarize the results of the included studies. The methodology must follow that of PRISMA or Cochrane systematic reviews.^15^ PRISMA is an evidence‐based minimum set of items for reporting in systematic reviews and meta‐analyses. PRISMA focuses on the reporting of reviews that evaluate randomized trials, but can also be used as a basis for reporting systematic reviews of other types of research, particularly evaluations of interventions.^19^

The difference between evidence‐based practice guidelines and systematic reviews relates more to the topic being reviewed and the scope of the review. Guidelines address an area of clinical practice and may have a large number of questions (>5) or statements to evaluate and collate in Summary of Findings tables. Guidelines provide explicit recommendations on clinical practice. Systematic reviews may address topics that are not directly related to clinical practice, for which there are a small number of questions (<5), or both. Systematic reviews might not provide explicit recommendations. The difference between the two approaches is not always clear and the methodologies have much in common.

## 4 METHODOLOGIST

We recommend including a methodologist who advises and guides the development to ensure appropriate rigor and consistency among these statements. This individual should be appropriately experienced in these consensus/summarization methodologies, but would not necessarily be a content expert. Primary responsibilities of the methodologist include the following:

- Ensuring that the panel adheres to established methodology.
- Keeping the development group focused on the scope and purpose of the AES.
- Assisting the Chair on all conference calls to ensure active participation of all group members and to prevent “group think” and other biases.
- Reviewing and editing survey questions.
- Limiting the results to statements of consensus or lack of consensus (for the Delphi method).
- Assisting with writing and editing the manuscript.
- Approving the final version of the manuscript for journal submission.
- Ensuring appropriate opportunities for disclosure of conflicts of interest and referral of management of COI as needed.
- Assisting with ensuring that consensus or lack of consensus is managed appropriately.

## 5 HOW IS THE TOPIC OF AN ACVIM‐ENDORSED STATEMENT DETERMINED?

Historically, the topic was chosen by the At‐Large BoR members after consulting with colleagues and solicitation of ideas from ACVIM Diplomates. Our opinion, after reviewing all of the ACVIM Consensus Statements published to date, is that the topics chosen have been timely and well‐balanced with respect to topical areas that are important to the broad interests of ACVIM Diplomates. Ideally methods to receive proposals for topics from the ACVIM should continue to be developed.

Oversight of the AES program will now be provided by the Education and Research Committee (ERC) of the ACVIM. We recommend that the ERC members, in consultation with the ACVIM BoR, develop a short list of proposed topics that then is posted on the ACVIM website, with the membership being notified and asked to respond within a specified period to rank and provide comments about the proposed topics, or make suggestions for additional topics.

The choice of topic will dictate the type of process used for AES development:

- The Delphi method is most useful for topics about which there is a limited amount of objective scientific evidence, or contentious topics about which there may be varied opinions but insufficient evidence to support development of a guideline.
- The GRADE approach is best for developing evidence‐based clinical practice guidelines for conditions with adequate amounts of objective scientific evidence.
- Systematic reviews are most appropriate for topics where there is a substantial amount of objective research regarding a limited number of questions.

The process for developing an AES would be to agree on the general topic and then for the ERC to seek advice from the President of the relevant specialty and the Chair of the panel on the most suitable format (ie, consensus statement, guidelines, or systematic review). The ERC and the relevant specialty President, guided by the methodologist, will be responsible for final approval of the AES topic and format.

## 6 WHO DEVELOPS THE ACVIM‐ENDORSED STATEMENT?

The AES should be developed by a panel comprised of individuals who are widely recognized as having expertise on the topic. Ideally, most panel members will have published basic research, clinical studies, or both on the topic or in closely related areas.

The process for identifying and selecting panel members should be carefully considered to ensure that selection is impartial, all potentially interested and qualified people have been identified and considered, potential conflicts of interest have been identified and effectively managed, the process for identifying panel members is accepted and actively supported by the ACVIM membership, and the final panel composition is approved by the ACVIM BoR, which may delegate this approval to the ERC. The process also likely will differ depending on the type of statement that is being developed (Table 2).

**TABLE 2:** Comparison and key features of ACVIM‐Endorsed Statements (consensus statements, evidence‐based practice guidelines, and systematic reviews).

	Consensus statements	Evidence‐based practice guidelines	Systematic reviews
Preferred methodology	Delphi method—consensus developed by experts for a series of statements using an explicit and iterative methodology	GRADE—structured, evidence‐based assessment. Can use GradePro software	PRISMA or Cochrane—methodologically rigorous review of published literature
Topic	Highly contentious topic with widely differing views among experts, limited scientific evidence	Addresses an area of clinical practice. Provides recommendations (evidence‐based) on one or more aspects of clinical practice	Suitable for meta‐analysis or detailed systematic review of topics that do not directly relate to clinical practice. Provides a considered, structured synthesis of available evidence for <5 key questions
Panel	2‐3 expert panelists who organize the process, and 11‐25 participants who provide expert opinions, supported by a methodologist. Each member is an expert in the general topic. A larger number of participants is an important feature of this process because of the more subjective nature, to ensure diversity of perspective	4‐6 expert panelists supported by a methodologist. Important to have a diversity of expertise that covers aspects of the topic (eg, clinician, pharmacologist, toxicologist)	2‐3 expert panelists, at least one of whom has experience in systematic reviews. Strictly objective review of literature is a foundational principle of Systematic Reviews, and allows for a smaller number of experts
Convening	Online, videoconferencing to facilitate discussion. Electronic voting on precisely framed questions	Online as required. Can use GradePro software	Online as required. Software tools such as Covidence are helpful to facilitate group participation
Level of evidence used	Limited numbers of objective studies are available to inform conclusions, and may provide less evidentiary power (eg, observational studies and expert opinion vs clinical trials)	Optimally, Practice Guidelines rely on availability of multiple systematic reviews and randomized clinical trials. However, lower levels of evidence are considered as needed to inform gaps in knowledge	High quality, objective studies that are reported as peer‐reviewed scientific publications
Consensus	Definitions of consensus established by organizers before participant meetings. Consensus/nonconsensus is determined for each item by voting of participants with reports of votes for each question. There may be more than one round of voting for any particular question	Objective assessment using GRADE criteria and reported for each question	Objective assessment of evidence using Cochrane or similar standards and reported for each question.
Outcomes	Reporting of consensus outcomes for a series of specific, precisely worded questions. The outcome is the result of the Delphi method, not reporting of evidence alone. The answers for which consensus is achieved identify opportunities to improve animal care and clinical outcomes	Evidence‐based recommendations taking into account the quality of evidence, strength of evidence, and feasibility and efficacy of treatments or interventions. Outcomes are the recommendations related to one or more of benefits, harms, values and preferences. A Summary of Findings table is important	Outcome is a critical evaluation of a series of specific questions. Outcome may include meta‐analysis. Suitable for topics that deal with, but are not limited to, epidemiology, pathophysiology, and public health
Reporting and public comment	Call for comment on topic and statements/questions before panel considers questions	Call for comment on topic and statements/questions before panel considers questions	Call for comment on topic and statements/questions before panel considers questions

### 6.1 Appointment of Chair

Panels for all types of AES will have a Chair who will be assigned specific responsibilities documented in a formal role description. The panel Chair should be an ACVIM Diplomate unless there are strong reasons to select a non‐ACVIM‐Diplomate for this role. The Chair and, if needed, Assistant Chair, of each panel will be selected after the topic of the statement has been finalized, and will be identified by consultation of the ERC with the relevant specialty President (if the topic is relevant to more than one specialty, then the Presidents of each of the relevant specialties will participate in the appointment). The ERC may seek additional advice from content experts and may call for nominations for the role(s).

### 6.2 Appointment of panel members

The process for appointing panel members differs for each type of expert statement. Typically, recommendations for membership are developed by the panel Chair in consultation with the ERC and President of the relevant specialty, with the assistance of the methodologist. The final composition of each panel is approved by the ACVIM BoR, which may delegate this approval to the ERC. At least half of the expert panel should be ACVIM Diplomates.

### 6.3 Consensus statements

For the Delphi method, the panel Chair, Assistant Chair, President of the relevant specialty and ERC will select 2‐3 panel members who will serve as core organizers and authors of the consensus statement. These panel members, with the support of a methodologist, then will identify the additional 11‐25 participants who will provide expert opinions that are the basis for content for the consensus statement. The methodologist should have expertise in the Delphi method and ensure that the correct approach and format are followed. The methodologist also will participate in writing the final document and ensure that submission deadlines are met. After a call for nominations (including self‐nomination), participants would be chosen from interested Diplomates who would need to provide evidence of their qualifications, including published research and clinical experience. Diplomates also could nominate qualified persons who are not members of the ACVIM (again, providing evidence of qualifications).

### 6.4 Evidence‐based practice guidelines

The panel Chair, Assistant Chair, President of the relevant specialty and ERC will select 4‐6 expert panelists who will work remotely to develop the guidelines, with the support of a methodologist. The methodologist should have expertise in the GRADE method and help ensure that the correct approach and format are followed. The methodologist also can participate in writing the final document and ensure that submission deadlines are met. After a call for nominations (including self‐nomination), participants would be chosen from interested Diplomates who would need to provide evidence of their qualifications (ie, published research, clinical experience). Diplomates also could nominate qualified persons who are not members of the ACVIM (again, providing evidence of qualifications).

### 6.5 Systematic reviews

When using PRISMA guidelines or the Cochrane methodology to conduct a systematic review, the ERC and President of the relevant specialty will select a content expert as panel Chair who then would recommend 2‐3 other content experts familiar with the development of meta‐analyses and systematic reviews to develop the statement. This approach also would benefit from the support of a methodologist familiar with meta‐analyses and systematic reviews.

## 7 PROCESS OF DEVELOPING THE STATEMENT

Regardless of the method used to identify the panel Chair and members, the Chair should drive the process, encourage equal participation by all panel members, coordinate the roles of the panel members, ensure diversity of opinion, drive the writing process to meet submission deadlines, format and editorial standards, and manage any identified conflicts of interest that become apparent (including potentially having a member with a relevant conflict of interest recuse themselves from the panel).

## 8 DEFINITION OF “CONSENSUS”

Where applicable, the definition of “consensus” must be based on criteria specified before the panel convenes to review evidence or prepare the content of their report. When a consensus statement is developed using the Delphi method, the planned number of rounds/iterations as well as criteria for stopping discussion should be specified before development of the consensus statement, and consensus will be defined as at least 75% agreement among authors.^18^ When the Guideline/GRADE approach is used, consensus or lack of consensus should be reported regarding rating of evidence quality and conclusions that can be drawn from that finding but is less impactful because recommendations are rated based on quality of evidence.^7^ When the systematic review methodology is used, reaching consensus is not generally necessary because recommendations are based on summarized, objective data.

## 9 REVIEW OF ACVIM‐ENDORSED STATEMENT

All AES will be made available to ACVIM Diplomates for review and comment before finalizing and publishing. The draft documents will be posted on the ACVIM website, available only to Diplomates, for online provision of comment and feedback. The document will be available for no less than 30 days and its availability will be announced to all ACVIM Diplomates by email. However, formal peer review of consensus statements or practice guidelines will not be conducted. At the discretion of the panel Chair and the JVIM Co‐Editors‐In‐Chief, it may be appropriate and desirable to obtain peer review for systematic reviews.

## 10 CONFLICT OF INTEREST

Results of a 2020 survey of ACVIM Diplomates regarding Consensus Statements shows that the ACVIM membership is keenly aware of the potential for conflict of interest (COI) to adversely affect the outcome and content of AES. It is critically important that such conflicts be identified and managed, and the Chair of the panel and the ERC, with support from ACVIM Staff, should be charged with this responsibility.

Simple disclosure of a potential COI does not represent management of that COI,^20^ and care must be taken that disclosure does not release the affected panel members and the panel Chair from further management or responsibility for the COI.

A COI is a set of conditions in which professional judgment concerning a primary interest (such as a patient's welfare or the validity of research) could be unduly influenced by a secondary interest (such as financial gain or career interests).^20^, ^21^, ^22^ Conflict of interest rules regulate the disclosure and avoidance of these conditions. A COI occurs when an entity or individual potentially becomes unreliable because of a clash between personal (or self‐serving) interests and professional duties or responsibilities. Such potential conflicts affect the ability to trust recommendations or the veracity of evidentiary statements when a company or person has a vested interest (such as money, status, knowledge, relationships, or reputation) that puts into question whether their actions, judgment, or decision‐making can be unbiased.^20^ Conflicts of interest can exist in the absence of any financial implications. When such a situation arises, the party is usually asked to recuse themselves. Importantly, perceived or potential COIs can tarnish the AES and the ACVIM unless clearly and transparently managed from the beginning of the process.

Important concepts in managing COI are identifying the potential COI (disclosure or discovery) and managing the COI.

### 10.1 Disclosure or discovery

To be managed, a COI must be identified. This can be done by either the individual (self‐disclosure) or by a third party (discovery). Although considered by some to be critically important to managing COI, there is limited evidence regarding the effect of COI disclosure. Research suggests that disclosure can have “perverse effects” or, at least, is not the panacea it is often perceived to be. These perverse effects include unwarranted immunity of the individual declaring the COI from criticism or censure for their role in developing an AES. Disclosure could have the unintended result of enabling or permitting a COI without preventing the effect of the COI.

### 10.2 Recusal or removal

Those with a COI are expected to recuse themselves from (ie, abstain from) decisions where such a conflict exists. It may be sufficient to recuse an individual regarding discussions and decisions of a specific topic, or it may be more appropriate for an affected party to be fully excused from participating in the AES. The obligation to recuse depends on: 1) the potentially conflicted individual identifying (ie, being aware of) and disclosing the potential COI, and 2) the individual recusing themselves (as opposed to a neutral party removing them from the situation). It could be appropriate for a neutral adjudicator to assess the potential COI and to decide on whether to retain or remove the individual in question.

### 10.3 Proposed process for identifying, considering and addressing potential or real conflicts of interest (based on those of the Endocrine Society^5^)

Optimally, AES panels would only include members who are free of COIs relevant to the topic of the statement. However, this will not always be feasible or possible, and processes must be in place to identify and manage COI. Transparency is key, and privacy concerns can be addressed by having the process widely acknowledged with details of the specific COI and management being private until publication of the statement, when COIs and management should be disclosed.

Processes for identification and management of COIs relative to the development of AES are summarized as follows:

- To be considered for membership on an AES panel, nominees are required to disclose all relationships that could affect or be perceived to affect the AES (and those of immediate family members) for the 12‐month period before the formation of the panel and any relationships that are entered into during development of the statement.
- Potential COIs that should be declared include all relationships with commercial, noncommercial, institutional, and patient or public organizations. These include employment, consultancy, interests in start‐up companies and those in which stock is not publicly traded, ownership interests (eg, stock options) in publicly‐traded companies (excluding indirect investments through vehicles such as mutual funds), research funding directly paid to the individual, research funding paid to an employer, organization or other research institution with whom the individual is involved, serving as a principal investigator, honoraria, royalties, paid or unpaid expert testimony, speaking engagements, speaker bureaus, and other relevant relationships. Leadership positions and memberships of other entities (paid and unpaid) including nonprofit or for‐profit advisory boards and committees must also be disclosed.
- It is critical that the panel Chair (and Co‐Chair or Assistant Chair) is free from COIs or potential COIs because they are responsible for managing potential COIs for the entire panel. For consensus statements using a Delphi process, it is more important for the organizing panel members to be free from COIs than for the other expert participants.
- Review of COI information is conducted as follows:
  - Panel Chairs and panel members: The ERC, the ACVIM Chief Executive Officer and the ACVIM President will review all COI information and determine whether any relationship represents a potentially relevant COI. The final recommendations on management of the COI are provided to the ACVIM BoR for information, endorsement and approval.
    - The Chair and Assistant Chair of the panel must be free of any COI or other biases that could undermine the integrity or credibility of the work.
    - A majority (>50%) of the panel members and participants in Delphi processes must be free of relevant COI.
  - Panel members with relevant potential COIs are required to declare the situation, and the ERC, Chief Executive Officer and President must determine whether the relationship represents a relevant COI and whether or not and how that COI should be managed. Management may include recusal from participation in all or part of the AES development process, including participation in relevant discussions, votes, and drafting recommendations.
  - Panel members are prohibited from adding new relationships that create COIs throughout the AES development process, until publication. If a new relationship is added, the ERC, Chief Executive Officer and ACVIM President must determine whether the relationship represents a relevant COI and whether or not and how that COI should be managed.
- If a relevant COI exists, it will be managed as follows:
  - Disclosure and Review:
    - The Chair of the ERC, with the assistance of the methodologist who is managing the AES, should interview the panel Chair and all proposed participants before initiating panel activities and ask the potential participants to disclose relationships that have the potential to create COIs.
    - If a panel member is aware that another participant might have a COI and has not declared it for some reason, they are obliged to bring this concern to the panel Chair's attention.
    - All potential COIs should be reviewed by the ERC, Chief Executive Officer and ACVIM President and any that require a management plan should be discussed with the affected participant.
  - Recusal:
    - Affected participants may be asked to recuse themselves from activities related to development of the AES for any part or all of the topics. Recused participants are prohibited from participating in developing or drafting guidelines sections that are directly related to their COI.
    - Recused participants are prohibited from determining the strength and direction of a recommendation directly related to their COI.
    - Recused participants are prohibited from voting on matters directly related to their COI.
  - Divestment:
    - Affected participants may alternatively choose to divest themselves of direct financial investments with entities that may have a potential financial interest in the contents of the guidelines or to otherwise alter or end relationships that are of a nonfinancial nature.
    - Panel members must also refrain from participating in the marketing activities or advisory boards of such entities.
  - Transparency:
    - The ACVIM will report details of all COIs that are identified as such by the ERC, Chief Executive Officer and ACVIM President in a detailed table that is included in the draft document reviewed by ACVIM members and in published guidelines.
    - More detailed COI documentation and management plans will be made available as needed in the form of published supplemental materials (to be available online).

## CONFLICT OF INTEREST DECLARATION

This editorial was prepared by the authors and was not peer reviewed. Kenneth Hinchcliff and Stephen DiBartola are Co‐Editors‐in‐Chief of the JVIM. None of the other authors declares a conflict of interest.
